# Low level DUX4 expression disrupts myogenesis through deregulation of myogenic gene expression

**DOI:** 10.1038/s41598-018-35150-8

**Published:** 2018-11-16

**Authors:** Darko Bosnakovski, Micah D. Gearhart, Erik A. Toso, Elizabeth T. Ener, Si Ho Choi, Michael Kyba

**Affiliations:** 1 Lillehei Heart Institute, University of Minnesota, Minneapolis, MN 55455 USA; 2 Department of Pediatrics, University of Minnesota, Minneapolis, MN 55455 USA; 3 University Goce Delcev - Stip, Faculty of Medical Sciences, 2000 Stip, Macedonia; 4 Department of Genetics, Cell Biology and Development, University of Minnesota, Minneapolis, MN 55455 USA; 5 Present Address: Research Center, Dongnam Institute of Radiological & Medical Sciences (DIRAMS), Busan, South Korea

## Abstract

Loss of silencing of the *DUX4* gene on chromosome 4 causes facioscapulohumeral muscular dystrophy. While high level DUX4 expression induces apoptosis, the effects of low level DUX4 expression on human myogenic cells are not well understood. Low levels and sporadic expression of DUX4 have been reported in FSHD biopsy samples and myoblast cultures. Here, we show that a large set of human myogenic genes is rapidly deregulated by DUX4, including *MYOD1* and *MYF5*, which are efficiently repressed even by low, non-toxic levels of DUX4. Human myoblasts modified to express low nontoxic levels of DUX4 were significantly impaired from differentiating into myotubes *in vitro*. Surprisingly, inhibition of differentiation does not require the transcriptional activation domain, thus is likely a feature of all mammalian DUX genes. DUX4 does not bind near the *MYF5* gene, but has a prominent ChIP-seq peak within the *MYF5* −118 kb enhancer. We find that when DUX4 binds at this site, it directs enhancer activity towards a nearby transcriptional start site for a noncoding nonfunctional RNA we name *DIME* (DUX4-induced MYF5 enhancer) transcript. These data highlight the anti-myogenic properties of DUX4 in human myogenic progenitor cells, and provide an example of enhancer disruption in the downregulation of *MYF5*.

## Introduction

Facioscapulohumeral muscular dystrophy (FSHD) is one of the most prevalent genetic myopathies^1^. It is caused by loss of repeat-induced silencing of a macrosatellite repeat at *4qter*, referred to as *D4Z4*^2–6^. Most commonly, silencing is disrupted due to reductions in repeat copy number^7^, but rare cases involve mutations of epigenetic regulators^6,8^. Each *D4Z4* unit encodes a copy of the *DUX4* gene, a transcription factor with two homeodomains^9^. Loss of silencing leads to *D4Z4* transcription, and the RNA transcribed from the last repeat unit is thought to be productively polyadenylated only in the context of a specific disease-associated allele downstream of the *D4Z4* repeats, which provides a poly-adenylation sequence^10,11^. Stable *DUX4* RNA then accumulates and is translated into the DUX4 protein, which then activates a number of target genes leading to deleterious consequences^12–14^.

A major problem for the field has been the inability to detect DUX4 protein in physical specimens from FSHD patients. Transcriptional profiling studies have inferred the presence of DUX4 protein due to its “fingerprint” of elevated target genes in FSHD biopsies^15^, however DUX4 must be rare, or expressed at low levels, or both, as fingerprint genes are expressed at extremely low levels in most FSHD samples (FPKM = 0 in many cases), and a consortium of genes is necessary to detect an effect, averaged over many samples. DUX4 can be detected in rare (on the order of 1/1,000) nuclei of cultured FSHD myoblasts^16,17^, and in their differentiated myotube derivatives at higher levels^18^. Forced high level DUX4 expression causes death of myoblasts and myotubes *in vitro*^12^, therefore it is possible that DUX4 is rarely and transiently expressed, leading to fiber loss. However sporadic and rare fiber loss is difficult to reconcile with the tremendous regenerative potential of human skeletal muscle and the total degeneration seen in certain FSHD-involved muscles. FSHD muscle does not pass through a highly regenerative phase on its the path to dystrophy, as does muscle in diseases associated with fiber damage due to impairment of the dystrophin glycoprotein complex (Duchenne, limb girdle, etc), thus a model in which regenerative potential is depleted through excessive cycles of damage and regeneration, such as is seen in these other dystrophies, lacks a strong foundation in the histological data.

Based on work in murine C2C12 myoblasts, we have previously proposed that regeneration is impaired in FSHD^12^. Very low levels of DUX4 expression, i.e. low enough that mouse myoblasts are not killed, will impair their differentiation into multinucleated myotubes *in vitro*, a result that has been replicated in primary cells from DUX4-inducible mice *in vitro*^19^, primary myoblasts transduced with DUX4^20^, and recently, in DUX4-inducible mice *in vivo*^21^. Although in humans, DUX4 expression levels are higher in differentiated myotubes than in proliferating myoblasts^18^, DUX4 is nevertheless detected in undifferentiated human myoblasts, for example by immunostaining in very rare cells mentioned above, and at levels that can activate expression of a 5xDUX4-binding site GFP reporter gene delivered by lentivector^22^. Since the frequency of GFP+ cells in these lentivector reporter experiments was more than an order of magnitude greater than the frequency of DUX4+ nuclei determined from immunostaining experiments, the DUX4 reporter assay must be more sensitive than DUX4 immunostaining. It is therefore reasonable to consider the possibility that many DUX4+ proliferating myoblasts are expressing low levels of DUX4 protein below the threshold for detection by immunostaining, levels that do not lead to cell death, but that may impair regeneration.

Whether human cells expressing low levels of DUX4 would show impaired differentiation potential like their mouse counterparts is not self-evident. This is because DUX4 induces overlapping but distinct sets of target genes in mouse and human cells^12,13^. The first transcriptional profiling study on FSHD myoblasts did indeed show reduced expression of MYOD1 target genes^23^. Interestingly, a recent meta-analysis of published human biopsy gene expression data has identified a gene set that serves as an FSHD signature of equal or greater strength compared to the DUX4 target gene signature, and this set comprises the inverse of the PAX7 downstream gene expression profile^24^. Because PAX7 is necessary in the skeletal muscle stem cell for maintenance of muscle regeneration, this profile suggests the possibility that, in spite of differences between human and mouse targets, DUX4 might impair the differentiation capability of human myogenic progenitors, as it does those of mouse. In the present study, we directly test this proposition, and find that DUX4 impairs differentiation of human myogenic progenitors; we discover the unusual mechanism by which DUX4 perturbs expression of the myogenic regulator, *MYF5*; and probe regions of the DUX4 protein necessary for inhibition of myogenesis in the human system.

## Results

### DUX4 perturbs myogenic gene expression

**Figure 1:**
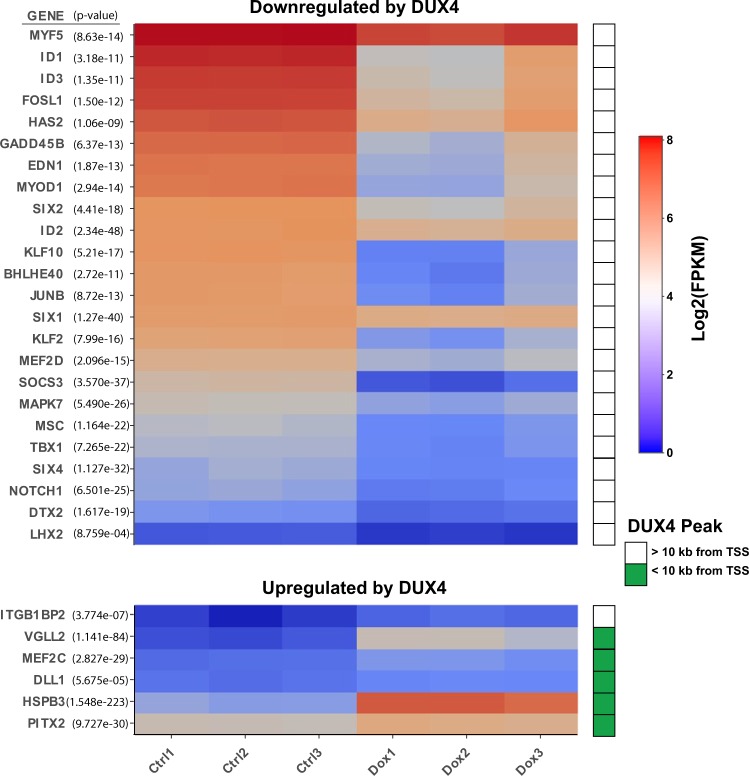
Myogenic genes perturbed by DUX4 within 6 hours. Triplicate RNA-seq profiles are rendered as a heat map. LHCN-M2-iDUX4 cells were induced for 6 hours with 250 ng/mL doxycycline. Downregulated targets are shown above; upregulated targets shown below. This rapid alteration in key myogenic regulators demonstrates that the myogenic program is deeply perturbed within 6 hours of DUX4 expression. The green box to the right of the heat map indicates genes with DUX4-binding sites identified by ChIP-seq within 10 kb of their TSS.

We previously engineered the immortalized human myoblast cell line LHCN-M2^25^ with a doxycycline-regulated DUX4 transgene to obtain the derivative cell line, LHCN-M2-iDUX4^14^, in which DUX4 can be induced to variable levels by treating cells with doxycycline at different concentrations. To identify the earliest gene expression changes induced by DUX4, we evaluated RNA-seq data from LHCN-M2-iDUX4 cells treated with doxycycline for 6 hours. In addition to a large number of upregulated genes, DUX4 provoked downregulation of a large number of genes, which is remarkable because both rapid cessation of transcription followed by turnover of previously expressed transcript would be necessary in order for downregulated targets to be identified within this short 6 hour time frame. Notable among these downregulated targets were the key myogenic regulatory factors, MYF5 and MYOD1. This prompted us to evaluate the global effects of DUX4 on the general myogenic program. Within the set of genes altered within the first 6 hours of DUX4 expression, we found that many important myogenic regulatory genes were strongly altered, in both directions – some being overexpressed, but the majority being repressed (Fig. 1). Because DUX4 is a transcriptional activator, the rapid downregulation of so many genes was unexpected. We therefore evaluated this list of perturbed myogenic genes for nearby peaks of DUX4 binding in LHCN-M2-iDUX4 ChIP-seq data. This revealed that the majority of upregulated targets had a DUX4 binding site within 10 kb of their transcription start site (TSS), whereas none of the downregulated targets had such a nearby DUX4 binding site (Fig. 1). This suggested that the downregulation of these many myogenic genes is not likely associated with DUX4-mediated recruitment of silencing factors, i.e. that DUX4 does not act as a direct repressor of transcription.

### Low level DUX4 expression in human myoblasts impairs MYOD1 and MYF5 expression and myotube differentiation

**Figure 2:**
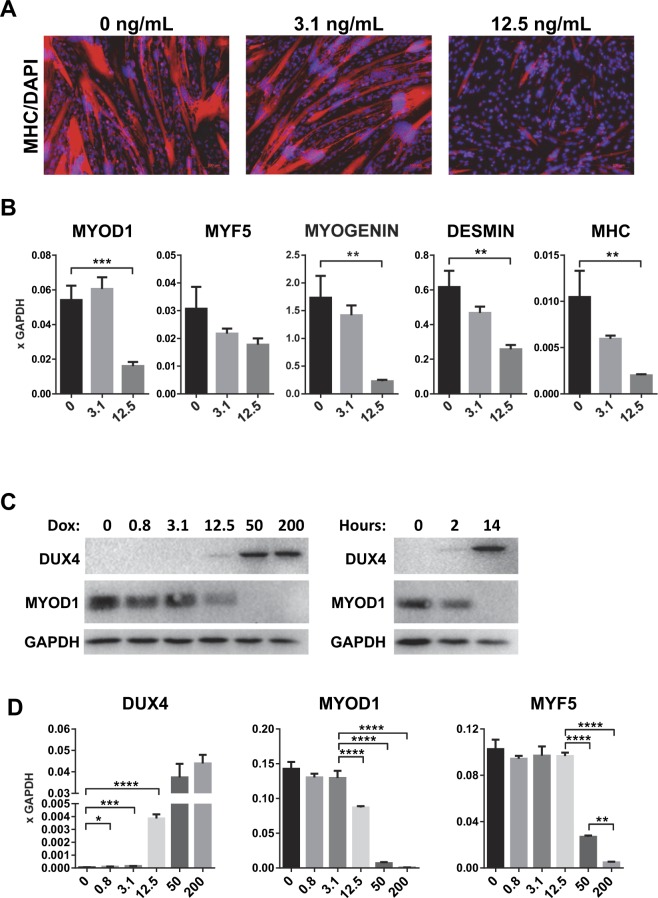
DUX4 inhibits myogenic differentiation. (**A**) Immunofluorescence for myosin heavy chain (MHC, MF20, red) on LHCN-M2-iDUX4 cells after 2 days of differentiation in the presence of 3.1 and 12.5 ng/mL doxycycline. Nuclei were counterstained with DAPI (blue). Scale bar 100 µm. (**B**) RT-qPCR for *MYOD1*, *MYF5, MYOGENIN, DESMIN*, *MYH3* (embryonic myosin heavy chain) on LHCN-M2-iDUX4 cells presented in (**A**) Data are presented as mean ± SEM; ***p < 0.001, ****p < 0.0001, T-test. Gene expression values are presented as fold difference to *GAPDH* (n = 4). (**C**) Western blot for DUX4 and MYOD1 on LHCN-M2-iDUX4 cells induced with various doses of doxycycline (dox) over 14 hours (left), and with 200 ng/mL doxycycline for 2 or 14 hours. (**D**) RT-qPCR for *DUX4, MYOD1* and *MYF5* on LHCN-M2-iDUX4 cells induced for 14 hours with various concentrations of doxycycline (ng.mL). Data represents mean ± SEM; ****p < 0.0001, ***p < 0.001, **p < 0.01, *p < 0.05 by one-way ANOVA with Tukey’s post hoc test. Results are presented as fold difference compared to *GAPDH* (n = 3).

To determine the effect of DUX4 expression on differentiation of human myoblasts, we exposed LHCN-M2-iDUX4 cells to a low dose of doxycycline, 3.1 and 12.5 ng/mL, to induce low levels of DUX4, which do not lead to cell death within 48 hours. While in the absence of doxycycline, LHCN-M2-iDUX4 cells could be differentiated into large, multinucleated, myosin heavy chain + myotubes, this differentiation was strongly impaired in the presence of 12.5 ng/mL doxycycline (Fig. 2A). This corresponded with reduced mRNA levels for *MYOD1*, *MYOG*, *DESMIN* and *MYH3* (Fig. 2B). It was previously shown that in mouse myoblasts, DUX4 rapidly downregulates both the RNA and protein levels of *Myod1*^12^. The gene expression changes shown in the heat map in Fig. 1 are at a dose of doxycycline that gives close to maximum output of the Tet-on system (250 ng/mL). To carefully investigate the activity of DUX4 against *MYOD1* mRNA and protein in the human system, we exposed LHCN-M2-iDUX4 cells to a doxycycline 4x dilution series, from 200 – 0.8 ng/mL doxycycline. DUX4 was detectable by western blot from 12.5 ng/mL, a dose at which MYOD1 was measurably reduced, both at the protein level and at the RNA level (Fig. 2C,D, Supplementary Fig. 1). At higher levels of induction, MYOD1 was undetectable by western blot, and reduced to near zero by RTqPCR. Because MYOD1 is a relatively short-lived protein, the time frame of protein loss was rapid, being virtually complete within 14 hours (Fig. 2C). The myogenic regulatory factors *MYOD1* and *MYF5* are differentially expressed in the myogenic hierarchy, but they show strong phenotypic compensation^26^. As *MYF5* was also downregulated by high level DUX4 expression, we performed a similar dose-response experiment to investigate regulation of *MYF5*. *MYF5* was measurably reduced at 50 ng/mL doxycycline and above (Fig. 2D).

### Inhibition of differentiation does not require the C-terminal 98 amino acid activation domain of DUX4

**Figure 3:**
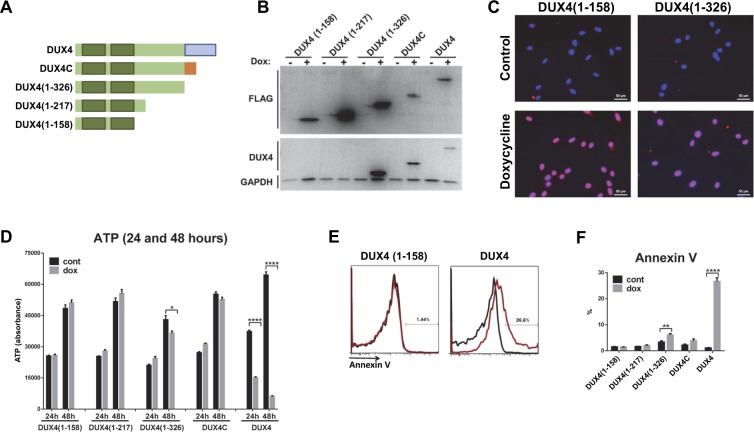
Cytotoxic effect of DUX4 deletion constructs. (**A**) Schematic diagram of the DUX4-ORF deletion constructs used for generating inducible human myoblast cell lines. Homeodomains are shaded green; transcriptional activation domain is blue, and the nonsense C-terminus of DUX4C is orange. (**B**) Western blot analyses of DUX4 deletion constructs after 14 hours induction with 200 ng/mL doxycycline (dox). All deletion constructs are labeled with a Flag epitope. Deletion proteins were detected with anti-Flag and anti-DUX4 antibodies. GAPDH was used as a loading reference. Note that the DUX4 rabbit monoclonal recognizes an epitope between amino acids 217 and 326, thus it does not recognize the smallest constructs. (**C**) Nuclear localization of DUX4 deletion constructs demonstrated by immunohistochemistry using Flag antibody in cells induced for 14 hours (dox 200 ng/mL). (**D**) Viability (ATP assay) of cells expressing various DUX4 deletion constructs after 24 and 48 hours induction with a high dose of doxycycline (200 ng/mL). Note that only full length DUX4 induces myoblast death within 48 hours. (**E**) Example of FACS profiles and percent of Annexin V positive cells in different cell lines after 18 hours induction (dox 200 ng/mL). (**F**) Quantification of Annexin V staining in various cell lines after dox induction.

**Figure 4:**
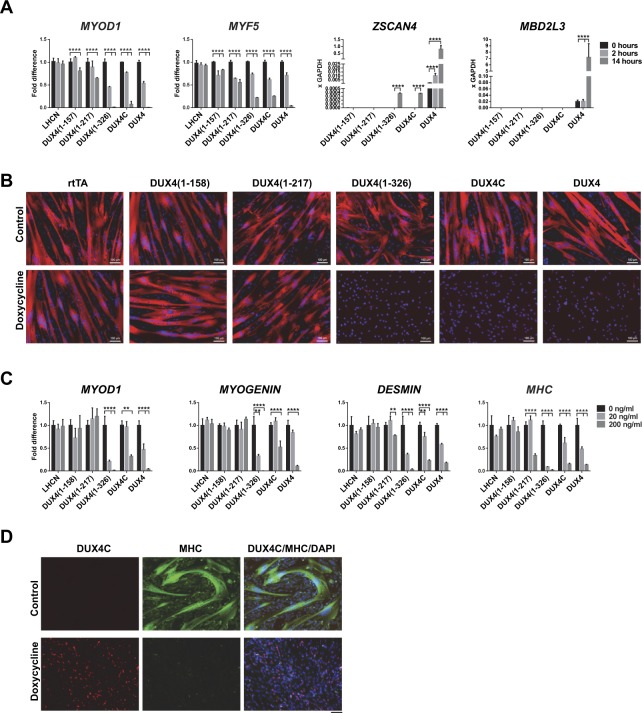
The DUX4 C-terminal activation domain is not necessary for inhibition of myogenesis. (**A**) RT-qPCR for *MYOD1*, *MYF5*, DUX4 target genes *ZSCAN4, MBD3L2* in cells induced with 200 ng/mL dox for 2 and 14 hours. (**B**) Immunostaining for MHC (red) after 2 days of myogenic differentiation. Nuclei are visualized with DAPI (blue) staining. DUX4 deletion constructs were induced with 200 ng/mL dox. (**C**) RT-qPCR for myogenic genes in cells represented in (**B**). Data are presented as mean ± SEM; **p < 0.01, ****p < 0.0001, by one-way ANOVA with Tukey’s post hoc test. Results presented as fold difference compared to control uninduced cells (n = 4). (**D**) Immunostaining for DUX4C (red), MHC (green) and DAPI (nuclei, blue) in LHCN-M2-iDUX4C cells after 2 days of myogenic differentiation and induction with 200 ng/mL dox. Note that DUX4C is expressed (red) in cells that were not able to terminally differentiate. Scale bar 100 µm.

The C-terminal 98 amino acid transcriptional activation domain of DUX4 is essential for its cytotoxicity. To determine whether it is also necessary for its inhibition of differentiation, we generated a number of mutant versions of DUX4, in which different lengths of C-terminus were lacking. We also tested DUX4C, a protein expressed from a satellite D4Z4 repeat that has a frameshift mutation replacing the C-terminal 98 amino acid activation domain with a nonsense C-terminus after amino acid 326 (Fig. 3A). It was shown in the mouse system that DUX4C also represses *MYF5*^27^ although the opposite result was shown in the human system^28^. These constructs were transduced into LHCN-M2 cells using the same dox-inducible lentivectors used to express full length DUX4. Expression of the constructs were confirmed by western blot and, and nuclear localization by immunofluorescence (Fig. 3B,C, Supplementary Fig. 2). We first tested cytotoxicity, and determined that all C-terminal deletions showed no or greatly reduced (DUX4[1–326]) cytotoxicity within 48 hours of induction (Fig. 3D). We tested induction of apoptosis directly by Annexin V staining, and found that no C-terminal deletion mutants were able to induce apoptosis, with the exception of DUX4[1–326], which showed a marginal but statistically significant increase in Annexin V staining (Fig. 3E,F). We also tested each construct for effects on transcription (Fig. 4A). By evaluating the canonical DUX4 targets, *ZSCAN4* and *MBD3L2*, it was apparent that only full length DUX4 can activate transcription effectively. The constructs lacking the terminal 98 amino acid activation domain, DUX4[1–326] and DUX4 C, showed only a marginally detectable increase in *ZSCAN4* expression, more than three orders of magnitude lower than the increase seen with full length DUX4. Interestingly however, all constructs showed some decrease of *MYOD1* and *MYF5* expression with DUX4[1–326] and DUX4C being about equal to full length DUX4.

We then induced this deletion panel of cell lines to differentiate (Fig. 4B). This showed that the C-terminal activation domain was not necessary for the inhibition of differentiation phenotype: both DUX4[1–326] and DUX4C potently inhibited the formation of multinucleated myotubes. However, the shorter constructs, having only the N-terminal half of the protein, had no discernable effect on differentiation. Evaluation of a number of genes associated with terminal differentiation further confirmed the phenotype (Fig. 4C). Coimmunostaining with MHC and DUX4C showed expression of DUX4C in cells that are not differentiating (Fig. 4D).

### DIME: a DUX4-Induced MYF5 Enhancer transcript

**Figure 5:**
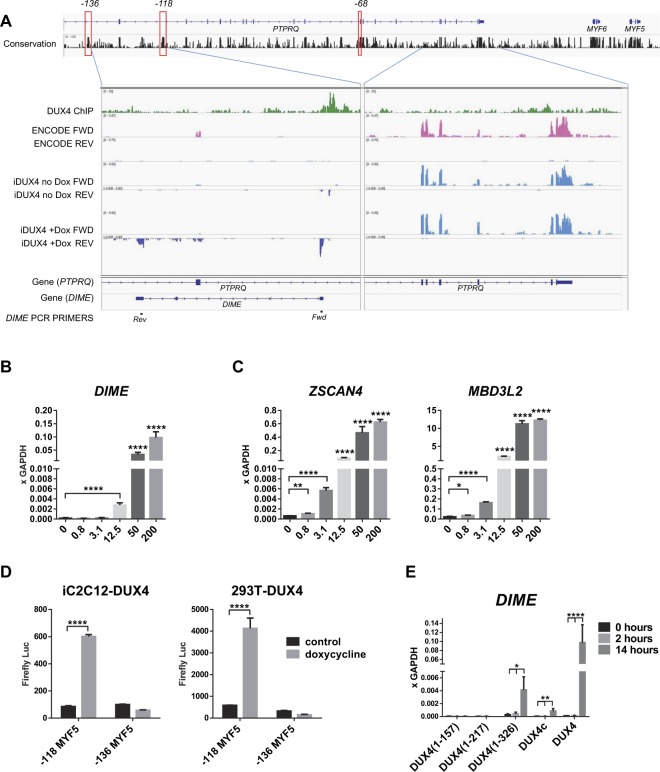
Effects of DUX4 expression on the *MYF5* −118 enhancer. (**A**) Map of the *MYF5* upstream region is shown above, with three defined upstream enhancers (−136 kb, −118 kb, and −68 kb, determined by LiftOver of the corresponding mouse −130 kb, −111 kb, and −57 kb enhancers, respectively) highlighted in red outline. Sequence conservation (score of the phastCons algorithm using alignment of 20 mammals) is shown below the gene model. ChIP- and RNA-seq blowups are shown below, zooming in on two areas: the left contains the −118 enhancer region, which has a DUX4 ChIP-seq peak and evidence of DUX4-induced reverse transcription; the right is a control region, lacking DUX4 binding, and showing only transcription in the forward direction (of the *PTPRQ* gene). DUX4 ChIP-seq reads^14^ are shown in green. ENCODE project RNA-seq in LHCN-M2 cells is shown in red (forward transcription above; reverse transcription below). Transcription of the exons of *PTPRQ* are apparent in the forward strand on the right panel. Below the ENCODE data is shown RNA-seq performed on LHCN-M2-iDUX4 cells in the absence of doxycycline, which shows a similar pattern to the parent LHCN-M2 cells. The last set of transcription tracks show LHCN-M2-iDUX4 cells exposed to doxycycline to induce DUX4. The forward strand expression of *PTPRQ* is still present, but the cells now show a new transcript adjacent to the −118 enhancer on the reverse strand, which we name *DIME* (DUX4-Induced MYF5 Enhancer), with two prominent exons. Gene models for *PTPRQ* and *DIME* are shown below, with qPCR primers designed for detecting spliced *DIME* transcript at bottom. (**B**) Expression of *DIME* transcript in LHCN-M2-iDUX4 cells induced with various concentrations of doxycycline for 14 hours. (**C**) Expression of the canonical DUX4 target genes, *ZSCAN4* and *MBD3L2*, in LHCN-M2-iDUX4 cells induced with various concentrations of doxycycline for 14 hours. (**D**) Luciferase assay in iC2C12-DUX4 and 293T-iDUX4 cells transfected with −118 kb or −136 kb MYF5 enhancer reporter constructs. Luciferase signal was measured 24 hours post induction (500 ng/mL doxycycline). Data are presented as mean ± SEM; *p < 0.05, **p < 0.01, ***p < 0.001, ****p < 0.0001, by one-way ANOVA with Tukey’s post hoc test (RT-qPCR) and t-test (luciferase assay). (**E**) Effect of various C-terminal deletion constructs on *DIME* transcription in cells induced with 200 ng/mL dox for 2 and 14 hours.

As mentioned above, neither *MYOD1* nor *MYF5* have nearby DUX4 binding sites. However, we discovered a DUX4 ChIP-seq peak within the −118 kb *MYF5* enhancer (Fig. 5A). The −118 kb enhancer corresponds to the −111 kb enhancer identified by transgenic studies in mice^29,30^. *MYF5* has a complex set of regulatory elements, most of which are distal to the neighboring gene, *MYF6*, actually within introns of the next gene along, *PTPRQ*. The DUX4 ChIP-seq peaks within the −118 kb *MYF5* enhancer colocalize with five putative DUX4 binding motifs. Interestingly, three of these cluster within a DNAse hypersensitive site in this cell line that is actually outside of the most highly conserved elements of this enhancer, while two sites cluster within the zone of greatest conservation (Supplementary Fig. 3). Looking more closely, we found that the DUX4 peak is associated with new RNA-seq reads on the opposite strand (Fig. 5A) suggesting that DUX4 is converting the −118 kb element from a distal enhancer for *MYF5* to a proximal promoter for a new transcript. We name this non-coding transcript the DUX4-Induced MYF5 Enhancer (*DIME*) transcript. The *DIME* transcript appears to be non-coding, lacking any ORFs, and thus probably non-functional. To test this, we cloned *DIME* from DUX4-induced cells, over expressed it in LHCN-M2 cells and found that it did not have any effect on *MYOD1* and *MYF5*, or on myogenic differentiation (Supplementary Figs 4 and 5).

To investigate the dose sensitivity of induction of the *DIME* transcript, we designed and employed an intron-spanning qPCR assay, which showed that *DIME* begins to show weak expression at 12.5 ng/mL dox, but shows quite strong expression at 50 ng/mL dox and above (Fig. 5B), the same point on the curve that *MYF5* expression is perturbed. Although it is strongly expressed, it is less sensitive than the most responsive DUX4 target genes, e.g. *ZSCAN4* and *MBD3L2*, which show a transcriptional response at 3.1 ng/mL dox (Fig. 5C).

Repurposing of the *MYF5*-118 enhancer to direct *DIME* expression rather than *MYF5* expression would be expected to cause *MYF5* expression levels to decline. To independently confirm DUX4-specific binding to the *MYF5-118* enhancer and to demonstrate functional activity, we designed luciferase reporter constructs that carry one of two known distal MYF5 enhancer elements: −118 kb and −136 kb. Interesting, only the −118 kb element showed functional activity in response to DUX4, as seen by elevated luciferase signal after 24 hours of DUX4 induction in iC2C12-DUX4 and 293T-iDUX4 cell lines (Fig. 5D). These data demonstrate that DUX4 can bind to, and activate transcription from, the −118 kb *MYF5* enhancer.

Interestingly, when we evaluated the C-terminal deletion constructs, we found that both DUX4[1–326] and DUX4C showed a measurable increase in *DIME* expression (Fig. 5E). Although this was less than WT DUX4, these constructs were much more potent at activating *DIME* than *ZSCAN4* or *MBD3L2* (Fig. 4A), WT DUX4 being about 25x more potent than DUX4[1–326] at activating *DIME*, while being >1,000x more potent at activating *ZSCAN4*, and even more so for *MBD3L2*. How can a version of DUX4 lacking its activation domain nevertheless activate expression of *DIME*? Given that *DIME* is adjacent to the *MYF5* −118 enhancer, it seems likely that DUX4 simply binding to this enhancer perturbs its normal regulation, allowing its activity to be inappropriately directed towards *DIME* transcription.

### DIME is induced strongly by DUX4 in myogenic cells

**Figure 6:**
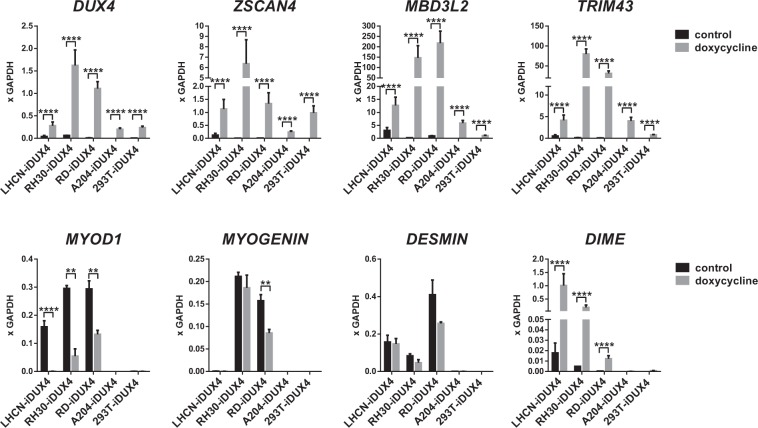
High level DIME expression by DUX4 is specific to myogenic cells. RTqPCR for *DUX4*, DUX4 target genes, myogenic genes and *DIME* transcript in DUX4-inducible human myoblasts (LHCN-M2-iDUX4), rhabdomyosarcoma cells (RH30-iDUX4 and RD-iDUX4), and non-myogenic (A204-iDUX4 and 293T-iDUX4) cell lines). Data are presented as mean ± SEM; *p < 0.05, **p < 0.01, ***p < 0.001, ****p < 0.0001, n = 4. Note that rhabdomyosarcoma cell lines express *MYOD1* and other related myogenic markers.

In order to understand whether *DIME* transcription in response to DUX4 is unique to the LHCN-M2 cell line, and whether it is more effective in cell types in which the *MYF5* −118 enhancer is active, i.e. myogenic cell types, we tested *DIME* induction by DUX4 in four additional cell lines. These included two rhabdomyosarcoma cell lines (RH30-iDUX4 and RD-iDUX4) and two non-myogenic cell lines (A204-iDUX4 and 293T-iDUX4). A204 is a human cell line established from sarcoma (originally thought to be a myosarcoma, but later revised), and 293 T is a human kidney cell line. We modified each cell line with the same dox-inducible DUX4 lentivectors used to generate LHCN-M2-iDUX4. We observed that the rhabdomyosarcoma cell lines expressed their signature markers *MYOD1* as well as downstream myogenic genes *MYOGENIN* and *DESMIN* (Fig. 6). In all cell lines, DUX4 robustly upregulated its canonical targets, *ZSCAN4, MBD3L2, and TRIM43* (Fig. 6). Similar to its activity in immortalized human myoblasts, DUX4 was able to downregulate *MYOD1* expression in the rhabdomyosarcoma cell lines. Remarkably, the DIME transcript was only transcribed at highly elevated levels in myogenic cell lines; in nonmyogenic cells, only very low levels of *DIME* transcript were induced by DUX4 (Fig. 6). Thus, we infer that *DIME* activation involves more than simply the DUX4 binding site, i.e. it seems likely that the −118 enhancer itself contributes to DIME expression through cooperative activity of enhancer-bound factors with DUX4.

## Discussion

These data demonstrate that DUX4 impairs myogenic differentiation in the human system. Similar results were observed when DUX4 was expressed in mouse myoblasts^12^, however the significance of that result was questioned because of the differences between the target genes of DUX4 in the mouse and human systems. Therefore, directly testing whether low non-toxic levels of DUX4 will affect human myogenic gene expression and impair human myogenesis is critical. Two additional findings are interesting in this regard: it was previously reported that FSHD myoblasts formed measurably thinner myotubes when subjected to differentiation^31^; and it was recently reported that the strongest gene expression profile detected in FSHD biopsies was not that of DUX4 up-regulated targets, but rather that of inverse-regulated PAX7 targets^24^. Both of these studies involving clinical material from FSHD patients lend support to the notion that myogenesis may be impaired in FSHD.

This impairment of myogenic differentiation by DUX4 is most likely due to the misregulation of a large number of myogenic genes, not to a specific single target. Because it is rapid, i.e. myogenic gene expression disruption is detected within 6 hours of DUX4 expression, the impaired myogenesis is not a secondary non-specific toxic effect of DUX4. In addition, certain non-toxic mutants of DUX4, for example deletion of the C-terminal activation domain, also impair myogenesis, providing strong support to the notion that the impaired myogenesis is not a secondary effect of DUX4 cytotoxicity. This correlates with recent findings that overexpression of the non-toxic protein DUX4C, which carries an alternative C-terminus due to a frameshift mutation, inhibits differentiation of mouse myoblasts^27^ and provokes cytoskeletal defects, and disorganization in human myotubes^32^. It is also notable that translocations of *DUX4* into the *IgH* locus have been implicated in B cell leukemia, and that all leukemia-associated translocations lack the 3′ region of the *DUX4* ORF, leading to overexpression of a version of DUX4 lacking the C-terminus^33,34^. This raises the possibility that inhibition of differentiation, probably through downregulation rather than upregulation of target genes, is a feature of DUX4 in multiple cell types.

Interestingly, there are three lineages of *DUX* genes in the mammalian genome, *DUXA*, *DUXB*, and *DUXC*^35^. *DUX4* is the human representative of the *DUXC* family^36^. Only the *DUXC* family has the transcriptional activation domain associated with cytotoxicity. Because the transcriptional activation domain and cytotoxicity in general are not necessary for impairment of differentiation, the *DUXA* and *DUXB* genes would also be predicted to inhibit differentiation.

Whether the effect of inhibition of differentiation by DUX4C could be responsible for some symptoms of FSHD is an open question. Although it has been reported to be expressed in FSHD^28^, it seems unlikely to be regulated by the D4Z4-distal polyadenylation signal thought to be causative of FSHD^11^. One possibility that bears investigation is whether there are sequence variants allowing *DUX4C* expression in linkage disequilibrium with 4qA alleles, as *DUX4C* is very near to the 161 SSLP associated with FSHD, which being proximal to D4Z4 is in fact actually much more closely linked to DUX4C than to the poly A signal sequence.

Because of the large number of DUX4-disrupted myogenic genes and the fact that they are not hierarchically related, impairment of myogenesis is likely multifactorial, i.e. not reversible by simple overexpression of any one of the downregulated factors. Because the majority of DUX4-disrupted myogenic genes are downregulated, and the transcriptional activation domain of DUX4 is not necessary for myogenic inhibition, the data implies that DUX4 is capable of regulating gene expression via multiple mechanisms, at least one of which does not require its C-terminal 98 amino acid activation domain. Furthermore, because the downregulated genes do not have nearby DUX4 binding sites, the mechanisms of downregulation are likely to be indirect. In this study, we probe one specific gene, *MYF5*, and discover an unexpected and quite unique mechanism of indirect downregulation: interference with activity of a far upstream enhancer by binding and inducing spurious transcription within the enhancer itself. We find that DUX4 binds within the −118 kb enhancer of *MYF5*, and induces a transcript which we name the *DIME* (DUX4-Induced MYF5 Enhancer) transcript. The *DIME* transcript has no obvious open reading frame and is likely to be nonfunctional. We speculate that its main effect is simply the disruption of the −118 kb enhancer, resulting in disengagement from, and therefore downregulation of, *MYF5*. The co-involvement of the −118 kb enhancer in regulation of *DIME* is supported by the fact that strong *DIME* induction by DUX4 is specific only to myogenic cells (where the −118 kb enhancer is active). Additionally, we found that a mutant version of DUX4 lacking its activation domain was able to induce *DIME* transcription. Although full-length DUX4 is about 25-fold more potent at *DIME* activation than DUX4[1–326] (DUX4ΔC), this difference is much greater at other DUX4 target genes. For example, full-length DUX4 is approximately 1,000-fold more potent at activation of the classical DUX4 target gene, *ZSCAN4*, compared to DUX4[1–326]. The significant activity of DUX4ΔC on *DIME* transcription suggests that mere inappropriate binding to the enhancer is capable of appropriating its activity towards alternative nearby targets, such as *DIME*. In further support, it has been demonstrated that a single DUX4 binding site is insufficient to induce target gene expression at low concentrations (i.e. at single copy gene concentrations of reporter) but rather that DUX4 transcriptional activation is highly cooperative^37^. DUX4 target genes typically have multiple clustered DUX4 binding sites^13,38^. This suggests the likelihood that DUX4 bound to the −118 kb enhancer cooperates with other −118 kb enhancer-bound factors to induce the *DIME* transcript, i.e. that enhancer activity is appropriated towards *DIME*.

Taken together, these results demonstrate that DUX4 at low, nontoxic, levels interferes with human myogenesis by perturbing myogenic gene expression. We show that effects on myogenesis are primarily due simply to the DNA binding activity of DUX4, facilitated by functional homeodomains, and do not require the C terminal activation domain. Finally, we discover an unexpected interaction mechanism between DUX4 and *MYF5*, by which a *MYF5* distal enhancer is disrupted by DUX4 binding. This is the first evidence of direct interaction of DUX4 with a key myogenic regulator.

## Methods

### DNA constructs

DUX4 deletion constructs iDUX4(1–156) and iDUX4(1–217) were PCR amplified from p2lox-DUX4^12^ and DUX4C was amplified from p2Lox-DUX4C^27^. Forward primers incorporated a 3xFlag tag sequence. The *DIME* construct was amplified from cDNA obtained from doxycycline induced LHCN-M2-iDUX4 using the following primers: AGTAGATGTAAAGGAAGTTTG and TTAGTGGCTTAAAACAATACC. PCR fragments were cloned into NheI/NotI sites of pSam2-iDUX4-Flag-UBC-puro plasmid^14^ using In-Fusion HD cloning (Clontech) to generate pSam2-DUX4(1–217), pSam2-DUX4(1–156) or pSam2-DUX4C constructs. All constructs were confirmed by sequencing. Reporter constructs were generated by cloning PCR-amplified and gel-purified DNA fragments into XhoI/HindIII-digested pGL4, upstream of a minimal promoter driving firefly luciferase, via In-Fusion assembly (ClonTech). Enhancer elements were amplified with following primers: −118 MYF5F CTCGAGTGAGGTTTCTGATACCCTTGATTT and −118 MYF5R AAGCTTCATCTTTGGGAGCTTTGTTTGT, and −136 MYF5F CTCGAGAAGAGAGTACATCTGTTCCTGAAA and −136 MYF5R AAGCTTCCAGGCAAGAGTTATTTGTAACC. The −118 MYF5 enhancer used in the reporter assay is 2291 bp and the −136 MYF5 enhancer is 970 bp.

### Cell culture and generation of doxycycline inducible cell lines

Immortalized human myoblast cell lines were cultured in proliferation media (F10 (HyClone) supplemented with 20% FBS (PeakSerum, Ps-FB3, lot 293Q16), 2-mercaptoethanol 1x (GIBCO), 10^−9^ M dexamethasone (Sigma), 10 ng/mL bFGF (Peprotech)), Glutamax (GIBCO) and Penicillin/Streptomycin (P/S, GIBCO) at 37 °C in a 5% CO2 atmosphere. For myogenic differentiation, cells were cultured on gelatin (0.1%) coated dishes in proliferation media until 100% confluence, then cells were washed with PBS and differentiated using differentiating media (DMEM/F12 (Corning Cellgro), supplemented with 1X ITS (GIBCO), 1X NEAA (GIBCO), Glutamax and P/S.

Inducible cell lines were generated as described previously^14^. Briefly, lentivirus was produced in 293 T cells by transfecting lentiviral vector construct pSam2 and packaging and envelope plasmids, psPAX2 and pMD2.G. Viral supernatant was collected at 48 and 72 hours post-transfection and used for transduction of the parent cell line, LHCN-M2-rtTA, a derivative of LHCN-M2, a human immortalized myoblast line cell line^25^, that ubiquitously expresses rtTA^14^. Transduced cells were selected for puromycin resistance using 1 µg/mL puromycin (InvivoGen).

A204-iDUX4, RH30-iDUX4 and RD-iDUX4 cell lines were generated in the same way as LHCN-M2-iDUX4. First, they were transduced with FUGW-rtTA lentivector, which carries an iresGFP reporter into parent cell lines: A204 (gift from Dennis Wigle, Mayo Clinic, Rochester, MN), RH30 (SJCRH30 (CRL-2061) ATCC) and R (RD (CCL-136) ATCC). Two days post-infection, GFP positive cells were sorted by FACS and expanded. GFP positive cells were then transduced with pSam2-DUX4 and selected in puromycin. 293T-iDUX4^14^, A204-iDUX4, RH30-iDUX4 and RD-iDUX4 cell lines were cultured in DMEM/F12 supplemented with 10% FBS and P/S. iC2C12-DUX4 cells^12^ were cultured in DMEM (HyClone) and 20% FBS. Newly generated cell lines were tested for doxycycline gene inducibility by immunofluorescence, western blot and RT-qPCR. RH30-iDUX4 and RD-iDUX4 were confirmed to be rhabdomyosarcoma cell lines that express MYOD and do not express MYF5.

### Identification of gene expression changes

We previously generated RNA-seq and ChIP-seq data on LHCN-M2-iDUX4 cells exposed to doxycycline for 6 hours and controls not exposed, which are available at the Gene Expression Omnibus repository under accession code GSE78158^14^. Expression FPKM values were extracted from among the differentially expressed genes (Benjamini-Hochberg adjusted p-value < 0.05), log2 transformed and plotted using the R package Heatmaply. DNase hypersensitive site data for LHCN-M2 cells was obtained from the ENCODE project accession ENCFF001BVR.

### Antibodies, immunostaining and western blots

Immunostaining was performed on 4% paraformaldehyde fixed cells, treated with 0.3% Triton X and blocked with 3% BSA (all from Sigma). Primary and secondary fluorochrome conjugated antibodies were diluted in 3% BSA and incubated overnight at 4 °C or for 1 hour at room temperature.

Western blots were performed on proteins separated on 8% SDS-PAGE gels and transferred to PVDF membrane. Primary and secondary HRP conjugated antibodies were diluted in 5% skim milk in TBST and incubated overnight at 4 °C or 1 hour at room temperature. HRP signal was visualized using Pierce ECL western blotting substrate (Thermo Scientific).

The following antibodies were used: mouse anti-MHC (MF20, 1:20, Developmental Studies Hybridoma Bank), rabbit anti-MyoD (1:200, Santa Cruz), rabbit anti-DUX4 (RD2-47c, 1:50, R&D Systems), Alexa fluor 488 Goat Anti-Mouse, Alexa fluor 555 Goat Anti-Rabbit (1:500, Invitrogen), Flag M2 (Sigma), GAPDH-HRP (1:5000, GenScript), 4′,6-diamidino-2-phenylindole (DAPI, Invitrogen). Full western blot images are presented in the Supplementary Figs S1 and S2.

### ATP assay

Cell lines were plated in a 96 well dish (2000 cells/well) and the following day induced with 200 ng/mL doxycycline. ATP assays were performed according to the manufacture’s instructions (Promega) by lysing the cells with 100 µl ATPlite and analyzing luminescence on a POLARstar Optima Microplate Reader (BMG Labtech, Offenburg, Germany).

### Annexin V/7-AAD staining

Cells were plated in 12 well dishes (10,000 cells/well) in proliferation medium. Two days after plating, cells were induced with 200 ng/mL doxycycline for 18 hours. Attached cells were trypsinized and stained with Annexin V and 7-AAD using the APC Annexin V staining kit (BioLegend) and run on a FACSAria II (BD) with data analysis by FlowJo (FlowJo, LLC).

### Luciferase assay

Cells were plated into 96-well dishes and transfected with reporter constructs using TransIT-LT1 reagent (Mirus). 24 hours after transfection, DUX4 was induced with 500 ng/mL doxycycline. Luminescence was measured at 24 hours post-induction using the ONE-Glo Luciferase Assay System (Promega).

### Quantitative Real Time RT-PCR (RT-qPCR)

RNA was extracted using an RNA extraction kit (Zymo) and cDNA was made using 0.75 µg total RNA with oligo-dT primer and Verso cDNA Synthesis Kit (Thermo Scientific) following manufacturer’s instructions. qPCR was performed by using Premix Ex Taq (Probe qPCR, Takara) and commercially available probes (*ZSCAN4*, Hs00537549_m1; *MBD3L2*, HS00544743_m1; *MYF5*, Hs00271574_m1; *MYOD1*, Hs00159528_m1; *MYOGENIN*, Hs01072232_m1; *MYH3*, Hs01074230-m1 *DESMIN*, Hs00157258_m1; *DUX4*, Hs03037970_g1; *GAPDH*, Hs99999905_m1; *B2M* Hs00187842_m1) (Applied Biosystems). DIME transcript was detecting using SYBR Premix Ex Taq (Tli RNaseH Plus, Takara) and primers: F: GCAGCAACATAGCTTTCCATC and R: CCCTACTCACTTCCTCCAATTC. Gene expression levels were normalized to that of *GAPDH* and analyzed with 7500 System Software using the ∆∆CT method (Applied Biosystems).

### Statistics

Replicates are biological, except where indicated. Group data was tested for normality (Kolmogorov-Smirnov test) and differences between groups were evaluated by T-test (if not indicated), or two-way analysis of variance (ANOVA) followed by Tukey’s or Sidak’s post hoc tests as indicated, using Graphpad Prism software. Differences were considered significant at p-values of 0.05 or lower.

## Electronic supplementary material

Supplementary Information

## Data Availability

The data that support the findings of this study are available from the corresponding author upon reasonable request.
