# Supplementary material for: Low level DUX4 expression disrupts myogenesis through deregulation of myogenic gene expression
Source: Sci Rep. 2018 Nov 16;8:16957. doi: 10.1038/s41598-018-35150-8 (PMC6240038; doi:10.1038/s41598-018-35150-8)

Low level DUX4 expression disrupts myogenesis through deregulation of myogenic gene expression

Darko Bosnakovski[1,2,3], *Micah D. Gearhart*[4], *Erik A. Toso*[1,2], Elizabeth T. Ener[1,2], *Si Ho Choi*[1,2,5], *and Michael Kyba*[1,2]

[1]*Lillehei Heart Institute*, [2]*Department of Pediatrics, University of Minnesota, Minneapolis, MN 55455, USA*

[3]*University Goce Delcev - Stip, Faculty of Medical Sciences, Krste Misirkov b.b., 2000 Stip, R. Macedonia*

[4]*Department of Genetics, Cell Biology and Development, University of Minnesota, Minneapolis, MN 55455, USA*

[5]*Present Address: Research Center, Dongnam Institute of Radiological & Medical Sciences (DIRAMS), Busan, South Korea*

Corresponding Author:

Michael Kyba, PhD
University of Minnesota
Cancer and Cardiovascular Research Building, 2231 6th St. S.E.
Minneapolis 55455, MN, USA
Email: kyba@umn.edu
Phone: 612.626.5869
Fax: 612.624.8118

Running title: DUX4 inhibits myogenesis

Key words: facioscapulohumeral muscular dystrophy, DUX4, myogenesis

Supplementary Figure Legends

Supplementary Figure 1. Full-length blots from Figure 2C. Western blot for DUX4 and MYOD1 on LHCN-iDUX4 cells induced with various doses of doxycycline (dox) over 14 hours (A), and with 200 ng/ml doxycycline for 2 or 14 hours (B).

Supplementary Figure 2. Full-length blots from Figure 3B. Western blot analyses of DUX4 deletion constructs after 14 hours induction with 200 ng/ml doxycycline (dox). All deletion constructs are labeled with Flag epitope. Deletion proteins were detected with anti-Flag and anti-DUX4 antibodies. GAPDH was used as a loading reference.

Supplementary Figure 3. Features of the -118 kb *MYF5* enhancer. Top track (blue), DNase-Seq data for LHCN-M2 cells demonstrating a single DNase hypersensitive site. Second track (green) DUX4 ChIP-seq, with predicted DUX4-binding sequence motifs shown below. Initiation of the *DIME* transcript on the opposite direction is shown below, in red, and annotation of the DIME transcript is shown at the very bottom of the figure. The PhastCons track, indicating degree of sequence conservation in the human sequence compared to 99 other animal species, shows that the most conserved block of sequence within the -118 enhancer is a block to the 3' of the hypersensitive site. We therefore amplified the sequence indicated in the "-118 MYF5 enhancer" track as a blue bar, which included both the initiation of DIME transcription as well as the hypersensitive site and the most conserved element from within this region, and used this sequence for further study.

Supplementary Figure 4. Full sequence of the *DIME* transcript, amplified from cDNA of doxycycline-induced LHCN-M2iDUX4 cells.

Supplementary Figure 5. Overexpression of DIME transcript does not affect myogenesis. (A). RT-qPCR for *DIME*, *MYOD1* and *MYF5* in LHCN-M2iDUX4 human myoblasts during proliferation. LHCN-rtTA (parent cell line), LHCN-iDIME and LHCN-iDUX4 were induced with 200 ng/ml doxycycline for 14 hours (B). Immunofluorescence for MHC (red) after 3 days of differentiation of LHCN-iDIME. Cells were induced with 200 ng/doxycycline while were in

differentiation medium (C). RT-qPCR for myogenic genes in the cells presented in B. (D) Note that overexpression of DIME does not affect expression of myogenic genes during proliferation or differentiation of LHCN cells. Results are presented as fold difference compared to *B2M* Data represents mean ± SEM; ****$p<0.0001$, n=6.

Supplementary Figure 1

A

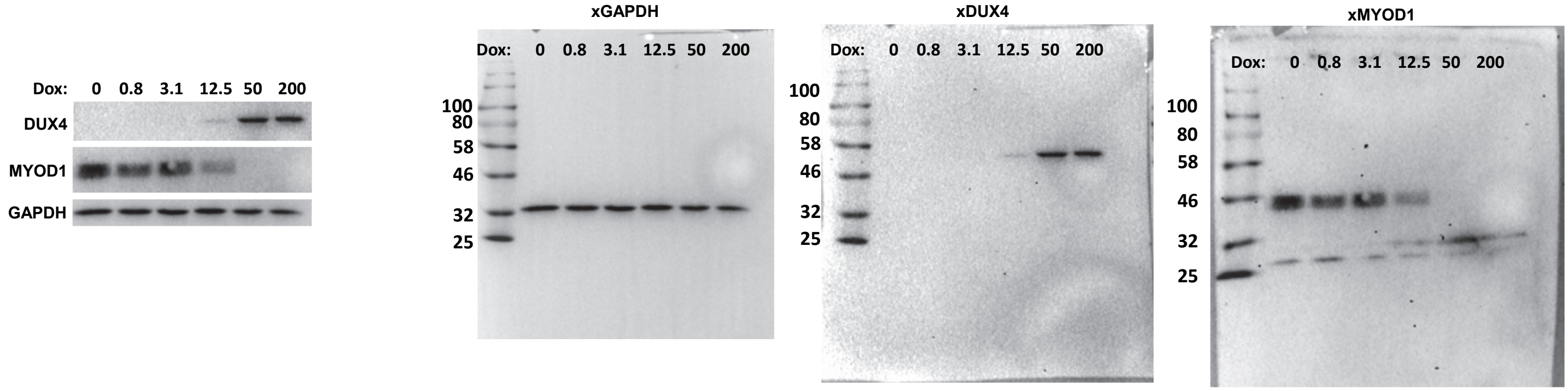

B

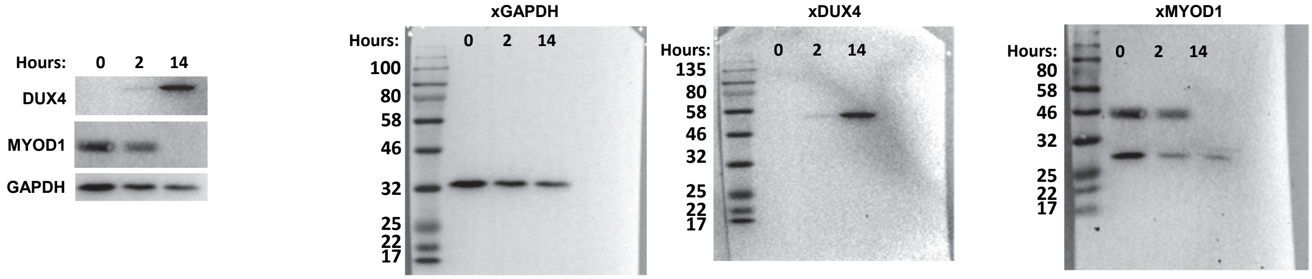

Supplementary Figure 2

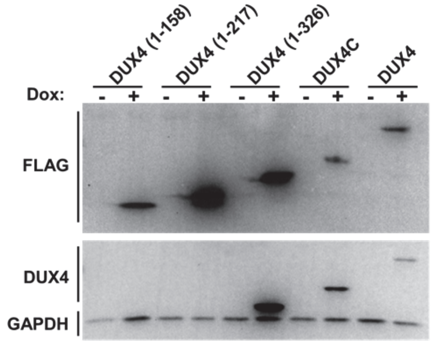

xFLAG (M2 antibody)

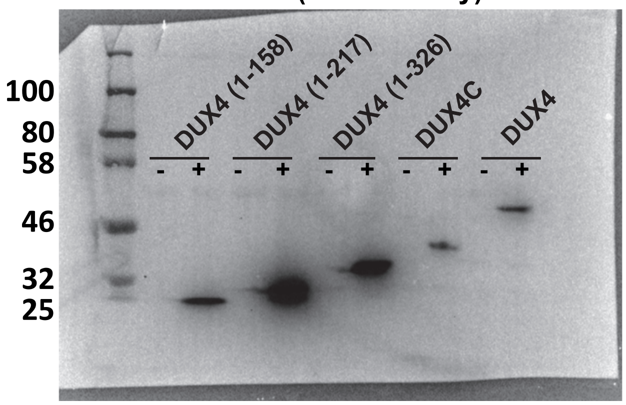

xDUX4 +xGAPDH

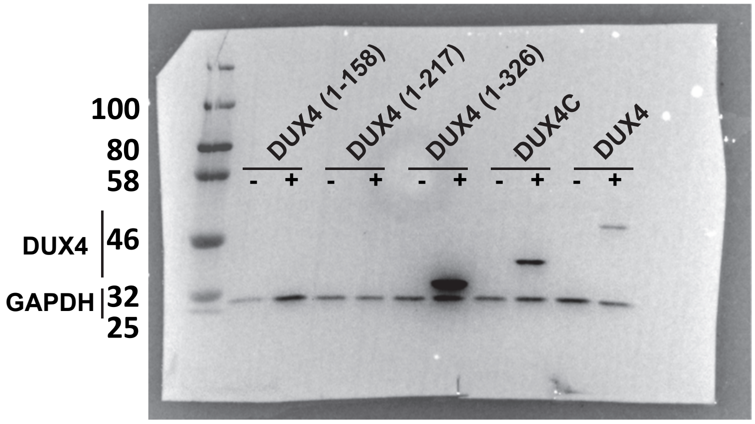

Supplementary Figure 3

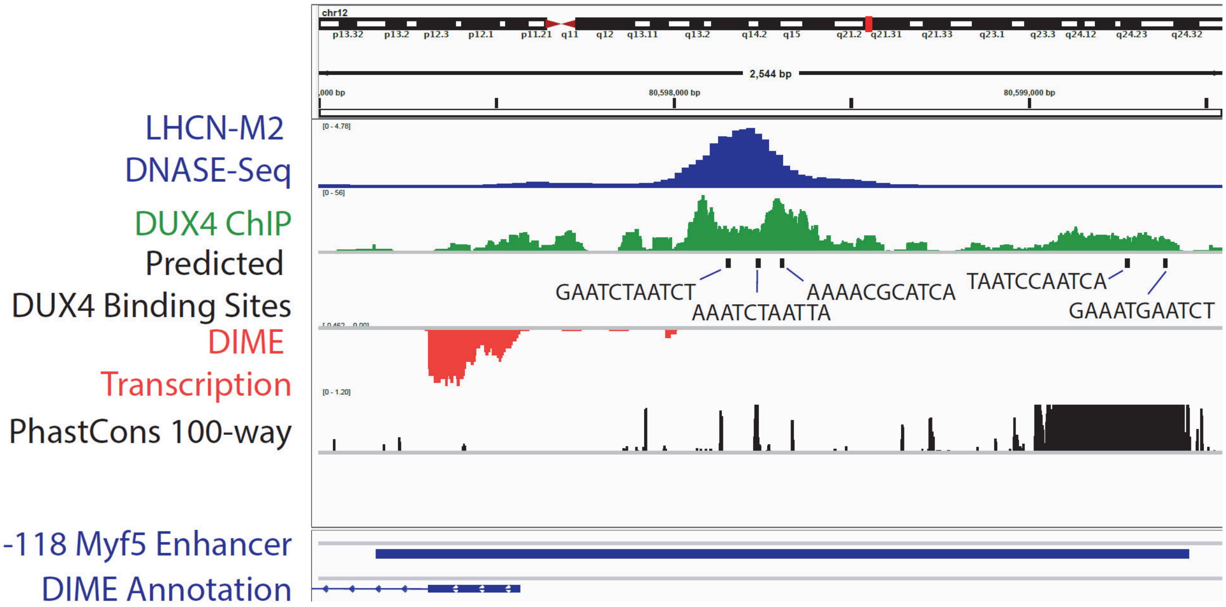

Supplementary Figure 4

Sequence of DIME transcript, amplified from LHCN-M2-iDUX4 myoblasts:

AGTAGATGTAAAGGAAGTTTGGGAGCTGGTAATCAACATGGAAACATGTATCAAGCCATCAAATATTCCACAGACTGGTCTCCTAT
CTTTCATTCTATTTTAATTCTGGATAAACTATGAAGAGGGTTCTCCCTACTCACTTCCTCCAATTCCCAAAGTTTTTAGAACAACAGG
TCAGCGTGTTCCATTAGAGCATAAAATATCACCATTTTCATGCTACCATGAAGCTCTGCATTGGAACAAACTGGAATAATTCAGATA
TCCCTCAAGATTCCCGGTCCCTGTACAAAAACAGATTCTTCCACTTACTCAACCAAACACTGATCTAGGTACAGATGGAAAGCTAT
GTTGCTGCTGTAACTAAGAATCTTAATCAGTTTACTTTAAAATAGAGAGCTATCCAGTGGACCTGACCTTATAACCTGAGCCCTCTA
AAAGGAGGATAGTATTTTTGGCCGTTTTCAGAAAAGGAAGTCAAAGCTTTAAAGAGATGGATTCAACGTACAAAAATTTCTCTAT
TGTTGGCTCTCAAGAAGGAGGGAGCTGCATGATAAGGAACACAGAACATCTTTGGGAGCTGAGAGAAGCCCTTAGCTAAAAGC
CAGCCAGGGAAGGGGCTCTCAATCCTGTGACTGCAAGGAACTAAATTCTACCACAACCACGTGAACCTGAAAGAGGACCCTGA
GTTACACAAGAAAATGTTGACTGACAAGTTGACTTCAGCTTGTGAGAATAAGTAGAAAACCTAGGCACAACACGCCTGGACTTC
TGACCTATAGAACTATGAGCTAATAAATGGGTATTGTTTTAAGCCACTAA

Supplementary Figure 5

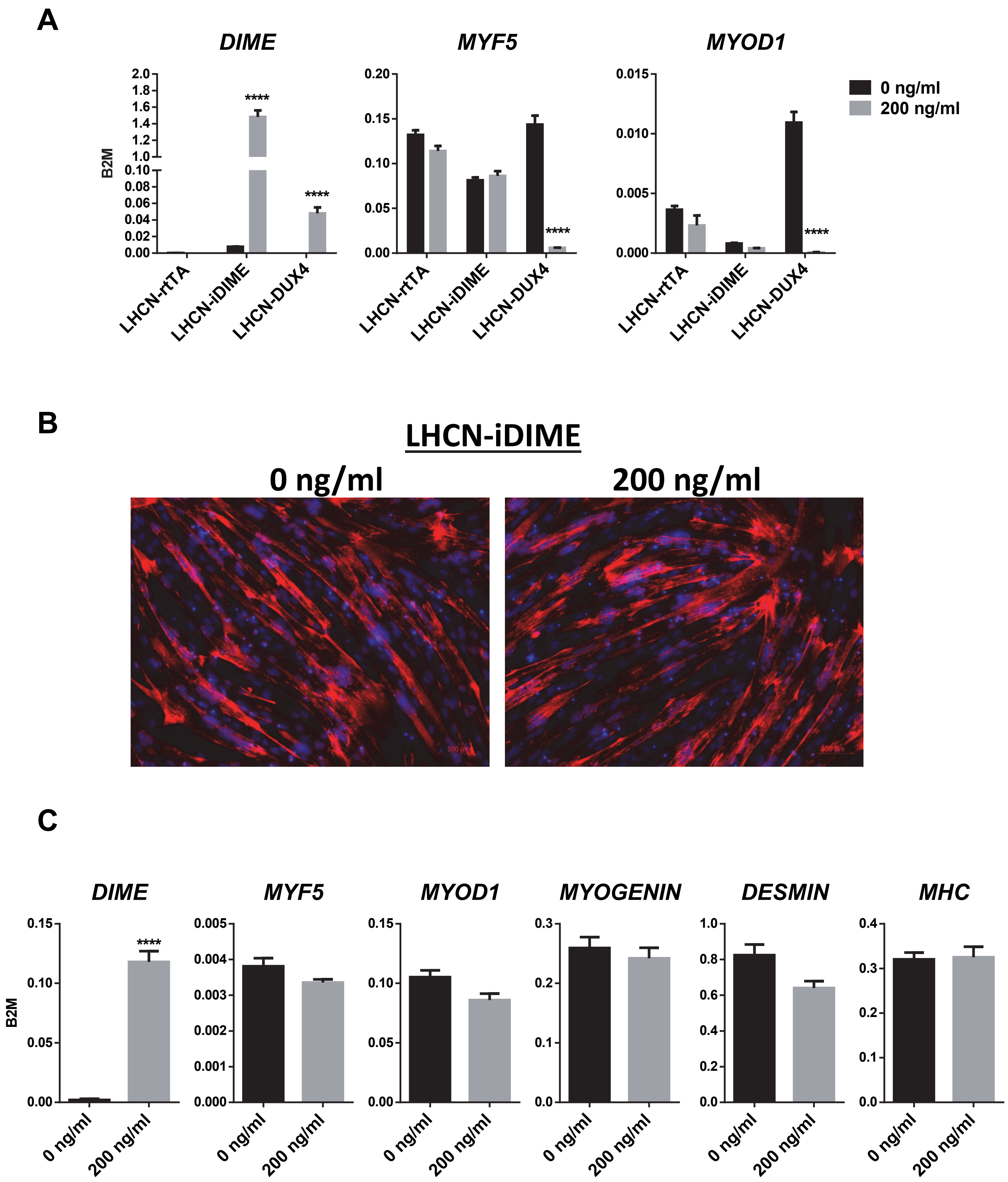

Supplement: Supplementary file 1 — Supplementary Information [file 41598_2018_35150_MOESM1_ESM.pdf]
